# Charcot osteoarthropathy in conservative treatment: clinical and functional results

**DOI:** 10.1186/1758-5996-7-S1-A17

**Published:** 2015-11-11

**Authors:** Jessica Castro de Vasconcelos, Rodrigo Gonçalves Pagnano, Mariangela Ribeiro Resende, Arnaldo Moura Neto, Denise Engelbrecht Zantut Wittmann, Maria Cândida Ribeiro Parisi

**Affiliations:** 1 UNICAMP, Campinas, Brazil

## Background

Charcot neuro-osteoarthropathy (CA) is a rare complication of neuropathy that affects patients who have lost protective sensation, has multiple etiologies and diabetes mellitus is the most prevalent. The CA is progressive degeneration of the affected joints and it is known that, in situations where there is not an adequate intervention, can install the complete destruction of the affected joints, as well as irreversible deformations, which lead to the development of ulcers and high index of amputation. In the consolidation phase, surgical treatment is usually indicated; however, the procedure is not always possible due to clinical limitations of patients, or even surgical difficulty itself. The suropodalic orthosis adapted to the patient may present as an alternative therapy.

## Objectives

To describe sample of patients with Charcot osteoarthropathy Eichenholtz (E) III, Showm (S) C, followed in diabetic foot outpatient clinic of a tertiary hospital in conservative therapy using suropodalic orthosis, evaluating as the main question the walking ability and accomplishment of daily tasks, and the occurrence of ulcer and/or infection.

## Materials and Methods

This is a prospective study, evaluated for the period of 5 years, 14 patients with CA, E III, SC, using suropodalic orthosis. Walking ability and accomplishment of daily tasks were routine annotated using as a tool of the domain 3 of the SF-36 (Medical Outcomes Study 36-Item Short-Form Health Survey), as well as occurrence of ulcer and/or infection.

## Results

**Figure 1:**
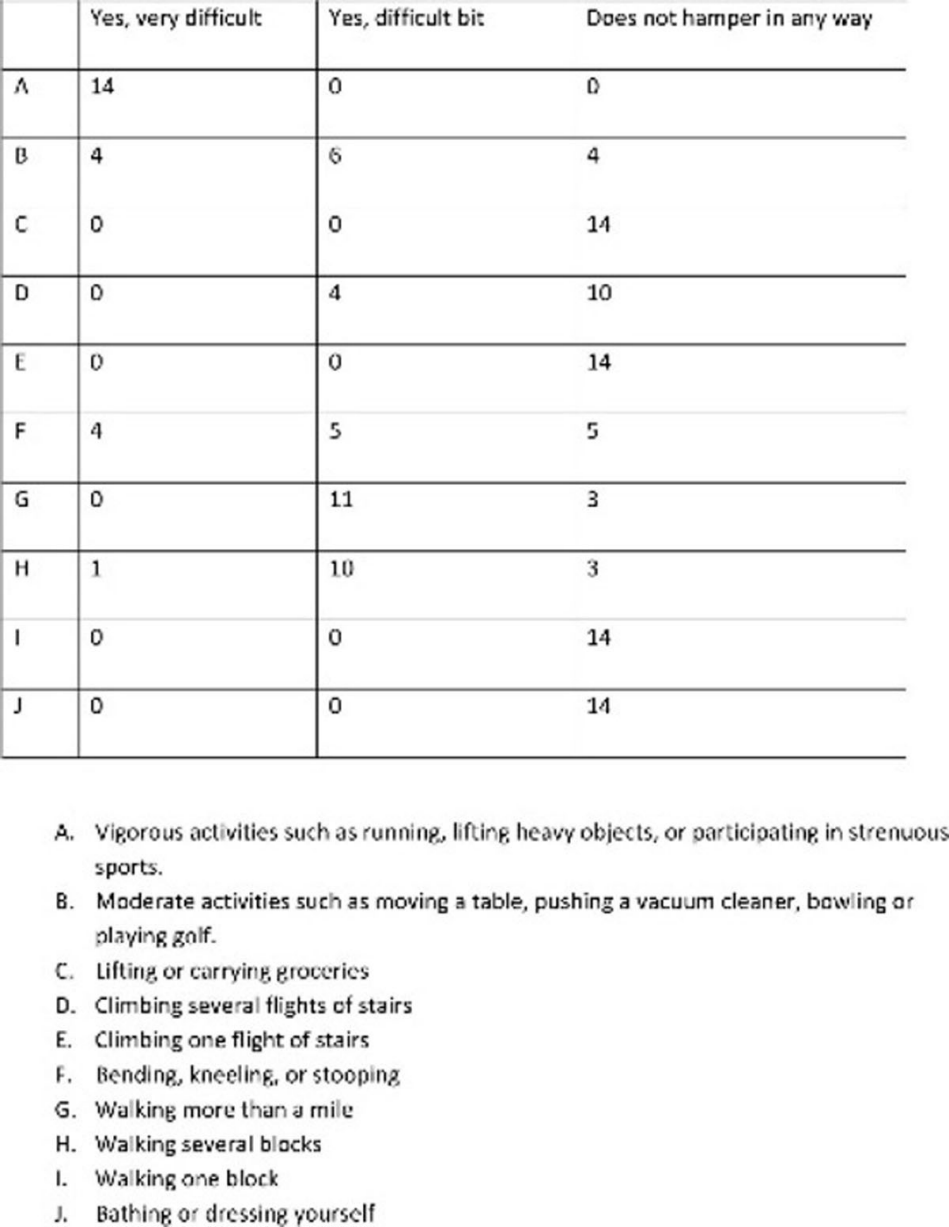
The following items are about activities you might now do during a typical day.

We evaluated 14 patients, 57% were women and 43% men, mean age 57.2 years, mean glycosylated hemoglobin 8.2%, with standard deviation of 2.69%. Diabetes average time of 10 years. In the evaluation of items related to function, walking and performing daily activities, we find satisfactory results (Figure 1). During the following five years, none of these patients had an episode of ulcer/or infection in the foot affected.

## Conclusion

Our data suggest that even in advanced stages of AC, when possible, the continued use of suropodalic orthosis may allow the maintenance function and walking ability.

